# Silver–Titania Nanocomposites for Photothermal Applications

**DOI:** 10.3390/gels11060461

**Published:** 2025-06-16

**Authors:** Leonardo Bottacin, Roberto Zambon, Francesca Tajoli, Veronica Zani, Roberto Pilot, Naida El Habra, Silvia Gross, Raffaella Signorini

**Affiliations:** 1 Department of Chemical Science, University of Padua, Via Marzolo 1, I-35131 Padova, Italy; leonardo.bottacin@unipd.it (L.B.); roberto.zambon.2@phd.unipd.it (R.Z.); francesca.tajoli@unipd.it (F.T.); veronica.zani@unipd.it (V.Z.); roberto.pilot@unipd.it (R.P.); silvia.gross@unipd.it (S.G.); 2 Consorzio Interuniversitario Nazionale per la Scienza e Tecnologia dei Materiali (INSTM), Via G. Giusti 9, I-50121 Firenze, Italy; 3 Istituto di Chimica della Materia Condensata e di Tecnologie per l’Energia (ICMATE), National Research Council (CNR), Corso Stati Uniti 4, I-35127 Padova, Italy; naida.elhabra@cnr.it

**Keywords:** sol-gel synthesis, green chemistry, nanocomposites, photothermal therapy, solvothermal treatment, Raman thermometry

## Abstract

Local temperature measurement is crucial for understanding nanoscale thermal transport and developing nanodevices for biomedical, photonic, and optoelectronic applications. The rise of photothermal therapy for cancer treatment has increased the demand for high-resolution nanothermometric techniques capable of non-contact intracellular temperature measurement and modification. Raman spectroscopy meets this need: the ratio of anti-Stokes to Stokes Raman intensities for a specific vibrational mode correlates with local temperature through the Boltzmann distribution. The present study proposes a novel photothermal therapy agent designed to advance the current state of the art while adhering to green chemistry principles, thereby favoring low-temperature synthesis involving limited energy consumption. A key challenge in this field is to achieve close contact between plasmonic nanosystems, which act as nanoheaters, and local temperature sensors. This is achieved by employing silver nanoparticles as a heat release agent, coated with anatase-phase titanium dioxide, as a local temperature sensor. The proposed synthesis, which combines refluxing and subcritical solvothermal treatments, enables direct anatase formation, despite its metastability under standard conditions, thus eliminating the need for a calcination step. Structural characterization through SAED-HRTEM and Raman spectroscopy confirms the successful crystallization of the desired phase. Moreover, the nanothermometry measurements conducted at various wavelengths ultimately demonstrate both the effectiveness of these nanomaterials as thermometric probes, with a relative sensitivity of about 0.24 K^−1^%, and their capability as local heaters, with a release of a few tens of degrees. This work demonstrates a new synthetic strategy for these nanocomposites, which offers a promising pathway for the optimization of nanosystems in therapeutic applications.

## 1. Introduction

Phototherapy constitutes one of the emerging methods for the removal of cancer cells from diseased tissues via irradiation of photothermal agents (PTAs) or photosensitizing agents [1]. In photothermal therapy, PTAs, which are characterized by high photoconversion efficiency, absorb energy in the form of photons and release it nonradiatively as heat. Thus acting as nanoheaters, they cause damage in the intracellular environment where they have accumulated to trigger apoptosis or programmed cell death [2].

The heating of the nanoheater must be sufficient to overcome the resistance threshold of cancer cells to hyperthermia. For this reason, it is often necessary to work in the range from 41 to 48 °C [3], or even above 50 °C, using lasers of high intensity, well-defined frequency, and pulse duration [1]. To uniquely identify target cells and avoid affecting even neighboring healthy cells or causing tissue inflammation, the heating process must be controlled, and the intracellular thermal distribution must be precisely monitored [4]. This can be achieved by employing nanothermometers with appropriate temperature detection ranges, high thermal, temporal, and spatial resolutions, with results independent of the concentration of PTAs, which allows measurements to be easily performed in a reproducible manner [4]. The detection of cancer cells is made possible by their temperature being higher than normal as a result of their enhanced metabolism.

The current state of the art shows a wide choice of experimental techniques used in order to detect the local temperature [5], which is why the potential of optical techniques to provide not only the surface but also internal temperature has been considered due to the ability of radiation to penetrate the biological window. Raman scattering spectroscopy has been proven to be one of the most appropriate as it allows for high spatial and thermal resolutions, which are necessary in the context of photothermal therapy [6,7].

The technique employed in this work to determine the local temperature of a material is Raman spectroscopy. Its common use in analyzing aqueous solutions, because of the weak Raman signals of water at low Raman shifts, makes it ideal for monitoring temperatures in a physiological environment. This method relies on acquiring both Stokes and anti-Stokes Raman spectra of the active material [8]. The ratio between anti-Stokes and Stokes Raman intensities varies with temperature because of the population distribution of vibrational states in the electronic ground state.

PTAs can work in the visible (400–700 nm) and near-infrared (NIR, 750–1350 nm) ranges. In the first, greater local heating is induced thanks to electronic resonances, while the second one allows greater tissue penetration since it falls in the biological optical window, which is transparent to radiation [1]. The temperature increases obtained in this way are lower but avoid tissue damage.

Many classes of organic and inorganic PTAs have been developed [9,10,11]. The most widely used are based on nanoparticles (NPs) suitably coated and functionalized to penetrate and selectively target cells. To cross the cell membrane via the mechanism of endocytosis, NPs must be of adequate size to be encapsulated in vesicles, typically 50–60 nm [12]. At the end of the photothermal treatment, it is then necessary to remove the NPs and whatever is left over as a result of apoptosis. Given the need to employ a nanosensor, it can be a separate particle from the nanoheater or incorporated into it [2]. In the first approach, there is no direct access to the temperature of the cancer cell because the nanoheater is placed at a certain uncontrollable distance from the nanothermometer. To retrieve it, heat transfer models are needed, but these are inaccurate at the nanoscale. For this reason, the second approach is preferable. The photothermal agent investigated in the present paper consists of Ag@TiO_2_ nanocomposite nanoparticles, where silver serves as the nanoheater and anatase coating serves as the nanothermometer.

Silver nanoparticles are widely studied for applications in various scientific fields [13], including nanomedicine, for antibacterial [14] and anti-cancer therapies [15], optoelectronics [16], and sensors [17,18], due to the possibility of modulating their physical, chemical, and biological properties according to different sizes and shapes.

Titanium dioxide, especially in its anatase form, stands out as an excellent candidate for Raman nanothermometry because of several advantageous features. It exhibits a strong Raman scattering cross-section and produces a prominent, easily distinguishable peak at low Raman shifts. These characteristics ensure clear and reliable measurements. Also, its low absorbance in both visible and near-infrared wavelengths minimizes sample heating from the laser source, enhancing measurement accuracy. It is highly chemically stable and non-toxic in biological settings, making it suitable for a wide range of applications, including those in biological contexts. Specifically, commercially available titanium dioxide nanoparticles have already proven effective as Raman-active nanothermometers in the visible spectrum, as demonstrated in previous research in both visible [19] and near-infrared biological windows [20]. Moreover, anatase nanoparticles synthetized using sol-gel methods possess properties comparable to commercial ones [21]. The ratio of the anti-Stokes and Stokes intensities of its intense Raman signal centered at ~145 cm^−1^ has been correlated with the local temperature of the sample and shows good reproducibility and sensitivity in a wide range of temperatures of biological interest (293–323 K).

Ag@TiO_2_ core–shell nanoparticles are typically synthesized using sol-gel methods. Titanium tetrabutoxide (TOB) is often preferred as the titanium alkoxide precursor due to its slower hydrolysis and lower cost [22]. Silver nanoparticle cores are usually formed by reducing silver salts, with the choice of reductant affecting the particle size and growth rate. Different sol-gel synthesis approaches have been developed, including chemical reduction [23], radiation-assisted reduction [24], and inverse micelle methods [25]. These can be further categorized into two-step methods (where pre-synthesized metal cores are coated) and one-pot methods (where core formation and shell growth occur simultaneously in the same reaction vessel) [26].

In the context of one-pot syntheses, various studies have explored different methods for synthesizing Ag NPs, with silver nitrate often being the precursor. Wang et al. utilized silver acetate and 1-dodecylamine for AgNP synthesis [27]. Liz-Marzán et al. used N,N-dimethylformamide with acetylacetone as a reductant and solvent [23]. Other researchers employed stabilizers like cetyltrimethylammonium chloride (CTAC) [28,29] and shell precursors such as titanium tetraisopropoxide (TTIP) and titanium-triethanolamine isopropoxide (TTEAIP) [25,30,31,32]. Zhang et al. produced hollow-core shells using NaCl to precipitate silver chloride [25]. Different reducing agents, including sodium borohydride and hydrazine, were used with stabilizers like CTAB [33,34,35,36]. Ti(SO_4_)_2_ was used in place of TTIP by Lin et al. [37], resulting in structures where the Ag nanoparticles were located at the edge of particles rather than in the middle, while Nithyadevi et al. found sodium alginate to yield more spherical cores [38]. Abdulla-Al-Mamun et al. proposed sodium citrate as a less toxic reducing agent and stabilizer for stable Ag@TiO_2_ suspensions [39].

A two-step approach allows for the production of nanoparticles in various shapes and sizes with improved monodispersion compared to one-pot syntheses [40]. Different precursors, such as AgNO_3_ and TOB, are used in the adopted syntheses, with variations in methods such as using TTIP to coat the core in the polyol procedure by Qi et al. [41] or forming a titanium glycolate shell in the method utilized by Yang et al. using TOB and Ethylen Glicole (EG) [42]. Other synthesis methods involve different reducing agents, like NaBH_4_ in the works of Kumbhar et al. [43] and Hong et al. [44] and hydroxylamine hydrochloride in the method used by Bartosewicz et al. [22], resulting in the formation of silver nanoparticles in a basic aqueous medium with observations of multicore–shells with core diameters of 20 nm or less.

In this study, both two-step and one-pot methods are employed for the synthesis of Ag@TiO_2_ nanocomposite materials, allowing for a comprehensive comparison of their respective advantages and outcomes.

The possible use of the Ag@TiO_2_ nanocomposite in photothermal therapy is optimally enhanced by the presence of a silver nanoparticle core, which acts simultaneously as a nanoheater and plasmonic substrate, and a shell of anatase, a biocompatible material, that acts as a Raman nanothermometer.

## 2. Results and Discussion

**Figure 1:**
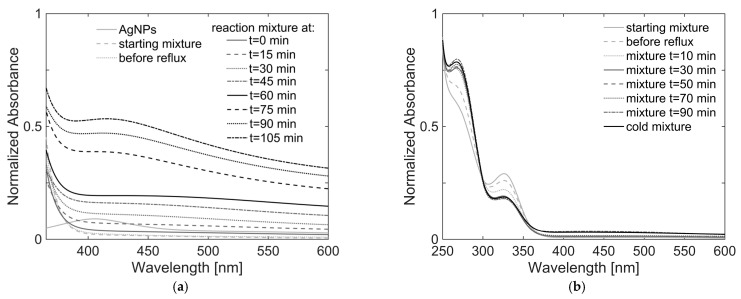
(**a**) UV/Vis spectra recorded every 15 min by taking small aliquots from the OP3 one-pot synthesis of Ag@TiO_2_ one-pot without dilution during its course. The spectrum of silver nanoparticles in DMF (*solution 2*) synthesized on the same day is shown for comparison (continuous light gray line). (**b**) UV/Vis spectra recorded every 20 min by taking small aliquots from the OP10 synthesis diluted.

Beginning with the established method of synthesizing silver nanoparticles in DMF [45], Liz-Marzán and colleagues introduced an additional step in coating these nanoparticles by carefully hydrolyzing titanium tetrabutoxide (TOB) and using acetylacetone as a stabilizing agent [23,32]. In this synthesis, the mixing sequence of the precursor solutions, i.e., *solution 1* (TOB/acetylacetone in ethanol) and *solution 2* (silver nitrate and water in DMF), within the reaction flask turned out to be of key importance. Titanium tetrabutoxide should be added promptly to the newly formed Ag NPs before they start to aggregate. One-pot reactions were carried out varying the molar ratios of titania to silver ranging from 5.0:1 to 6.2:1, obtained by varying the amount of AgNO_3_, in the range of 21–23 mg, and the volume of *solution 1*, from 20 to 100 mL and *solution 2*, from 5 to 25 mL; a complete and detailed overview of all samples prepared (called OP1-OP11) and the exact quantities used are reported in SI (Table S1). All syntheses showed the same qualitative behavior when monitored using UV/Vis spectroscopy. As an example, the course of the OP3 reaction monitored every 15 min is shown in Figure 1a, along with the spectrum of Ag NPs *solution 2*, while the spectra of the diluted samples of OP10 synthesis, taken every 20 min, are presented in Figure 1b.

The spectrum of the mixture immediately after the sequential addition of the two precursor solutions (dotted light gray line in Figure 1a) shows no plasmonic band attributable to the silver nanoparticles. This is understandable considering the strong dilution imposed on *solution 2* (continuous light gray line), following the addition of ethanol, which was four times the volume of DMF. However, at wavelengths shorter than 360 nm, strong absorption due to the titania precursor solution is evidenced (clearly shown in Figure 1b). Since the reaction mixture was refluxed, a progressively more intense band at 417 nm is observed. Compared with the spectrum of *solution 2*, whose maximum falls at 405 nm, it can be concluded that the observed band is due to the presence of TiO_2_ around silver core NPs. The redshift of Δλ = 12 nm is explained by the change in the refractive index of the medium surrounding the Ag NPs due to the presence of TiO_2_. The plasmonic resonance is generally in the range of 416–480 nm, as confirmed by literature works [31,46,47].

Under dilution conditions of sample OP10, it is possible to appreciate how its plasmonic signal growth is quite similar to that of OP3 and continues even after the reaction is over, until it cools to room temperature (Figure 1b). In the lower wavelength range, with respect to the plasmonic peak, the two bands of acetylacetone and TOB acetylacetonate are mainly visible at 269 and 327 nm, respectively. During the formation of the shell around the Ag NPs, the intensity of the band of acetylacetone increases, while that of the titanium (IV) acetylacetonate complex decreases. This seems to suggest the removal efficiency of the chelating agent by water under refluxing conditions, thus allowing for a controlled process of hydrolysis. By comparing the spectra of all samples, no clear relationship was found between the intensity and the position of the plasmonic band and the TiO_2_:Ag molar ratio used.

Regarding the amorphous OP suspensions obtained, DMF had to be removed before placing them in a solvothermal bomb via centrifugation and washing with ethanol. Purification operations proved to be successful, which also led to the removal of free residual acetylacetone. The following solvothermal treatment, performed at 150 °C for 24 h on the various samples, led to different results in terms of suspension coloration, which varied between colorless, sand-brown, and black (see table in SI). In particular, only the OP4 and OP8 syntheses showed a light sand-brown coloration.

**Figure 2:**
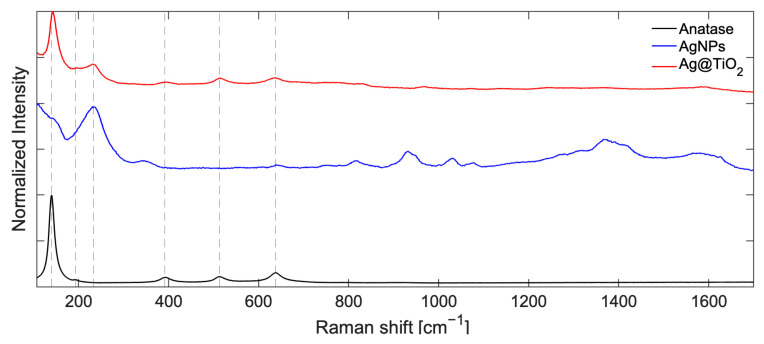
Comparison of normalized Raman Stokes spectra of OP4 (red line), Ag (blue line), and TiO_2_ (black line) nanoparticles between 107 and 1700 cm^−1^, recorded with excitation at 514 nm. Dotted lines are only guide for eyes.

**Figure 3:**
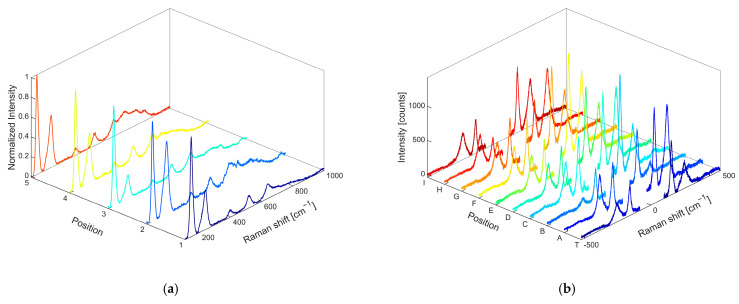
Raman spectra of OP4. (**a**) Stokes spectra of different positions of the sample (1–5), excited at 514 nm and normalized against the anatase signal at 143 cm^−1^. (**b**) Anti-Stokes and Stokes Raman spectra of the different positions (A-I,T), excited at 800 nm. Raman spectra are recorded at input power of about 1.0 mW.

The first confirmation of the formation of nanocomposite systems was provided by Raman measurements performed at 514 nm on Ag and TiO_2_ NPs, as well as on the synthetized nanocomposites, as shown for OP4 in Figure 2. The signal due to Ag-O, visible on the blue line, is centered at $\tilde{\upsilon }=241\;cm^{-1}$ [48,49,50,51], while the characteristic peaks of anatase, black line, are found at the frequencies of $\tilde{\upsilon }=148,399,516,635\;cm^{-1}$, which correspond to $E_{g}\left(1\right),B_{1g},A_{g}+E_{g},E_{g}(2)$ (the spectrum of commercial anatase is also reported in the SI, Figure S2, for comparison). Features ascribed to silver and titanium oxide, the latter occurring as a crystalline phase of anatase, are present in both the OP4 and OP8 nanocomposites. To make sure that anatase was present throughout the sample, different positions of the dried powder were analyzed by showing the expected core and shell signals everywhere, leaving out the relative intensity of the signals. The Raman spectra obtained from different positions of the OP4 sample, excited at 514 nm, are reported in Figure 3a: it is possible to clearly show both anatase and Ag oxide signals in different positions of the sample. The OP4 sample was also further mapped in the near-IR range at the wavelengths of 800, 850, and 900 nm. The mapping results, collected at 800 nm, are shown in Figure 3b, where the Raman spectra collected at different positions (indicated by the letters A–J, T) are shown.

**Figure 4:**
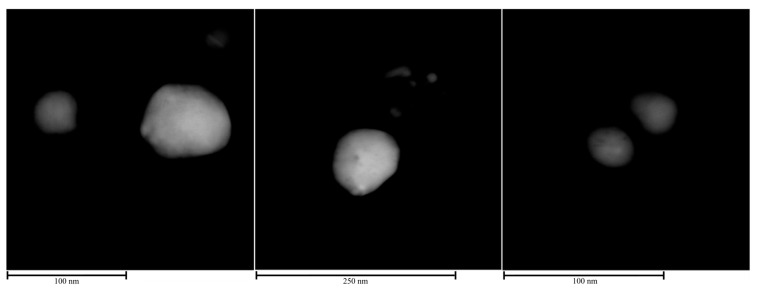
HRTEM dark field images of three different positions of OP4 sample.

**Figure 5:**
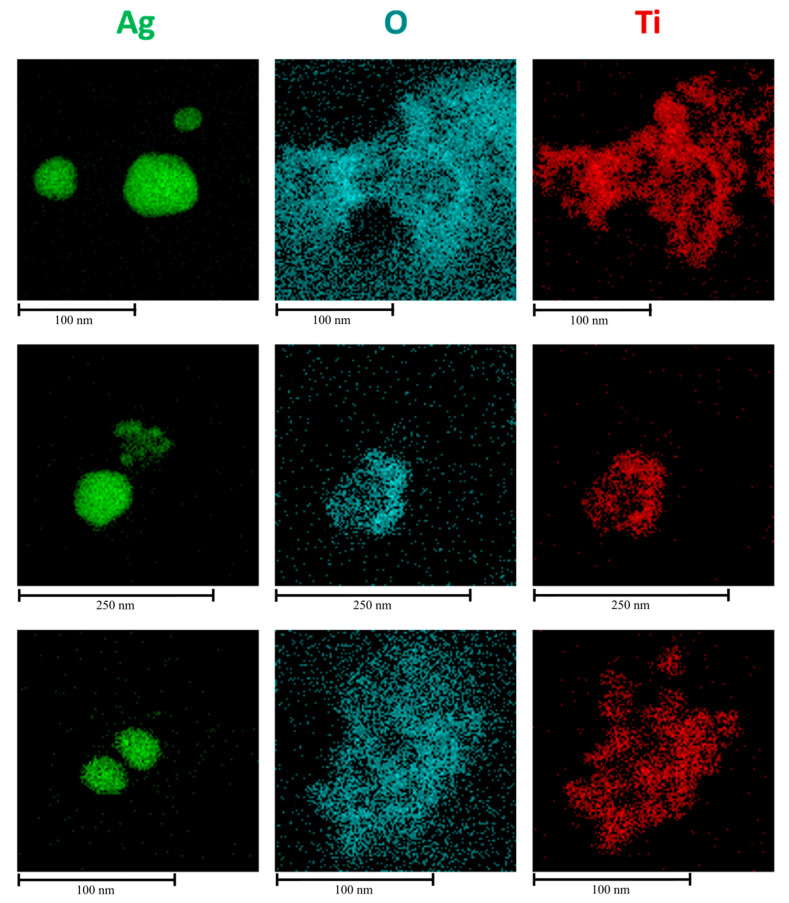
EDS maps for the three regions explored by HRTEM reporting silver (green), oxygen (blue), and titania (red), respectively.

The definitive confirmation of the presence of a crystalline shell around the silver nanoparticles was obtained from HRTEM analysis coupled with EDS, as reported in Figure 4 and Figure 5, respectively. Three different regions of the TEM grid where the suspension was deposited were analyzed and shown in dark field in Figure 4. The EDSs reveal that titania anatase forms a kind of diffuse coating around Ag NPs (Figure 5), which is precisely an indication of the formation of a nanocomposite nanoparticle using the one-pot method.

Upon examination, the nanocomposite exhibits a striking degree of heterogeneity in terms of particle morphology and dimensions. The nanoparticles present in the sample display a wide range of sizes, with a notable disparity between the largest and the smallest entities. The most substantial particles observed have diameters reaching approximately 100 nm, while the smallest detectable particles measure around 30 nanometers in diameter. This significant size distribution, spanning 30 to 100 nm, indicates a lack of uniformity in the nucleation and growth processes of these nanoparticles.

With the aim of gaining more control over the size of the nanosystems and replacing the non-green reducing agent, such as DMF, with green ones by working in aqueous or ethanol solutions, the possibility of realizing the same systems through a multistep sequential synthesis was explored. In this case, the synthesis of small Ag NPs was followed by a step of controlled growth of the silver cores and their coating with the titanium oxide layer.

**Figure 6:**
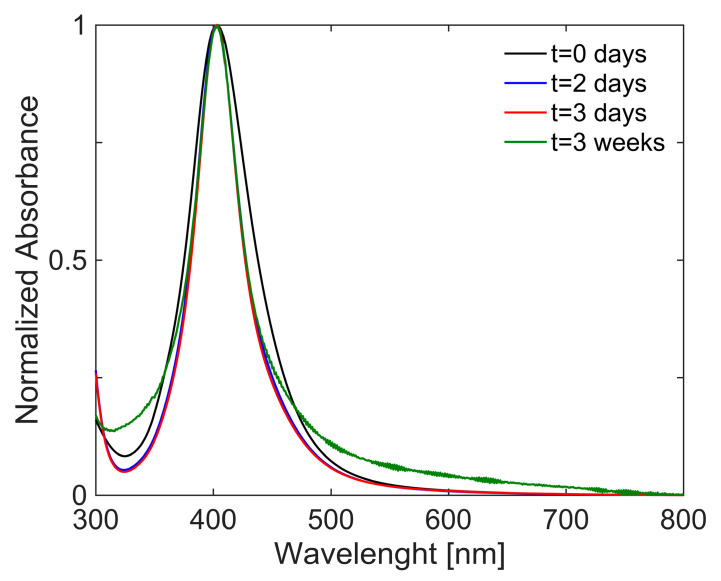
UV/Vis spectra of Ag seeds suspension at t = 0 (black line) and during the three weeks after the synthesis: t = 2 days (blue line), t = 3 days (red line), and t = 3 weeks (green line).

The seed suspension exhibited a sharp absorption band centred at 403 nm, reported as a red line in Figure 6, which is indicative of the presence of Ag NPs with an average diameter of ~30 nm [52]. The seed suspension is characterized by great stability over time, which was evaluated by comparing spectra acquired over a period of three weeks after synthesis (Figure 6).

**Figure 7:**
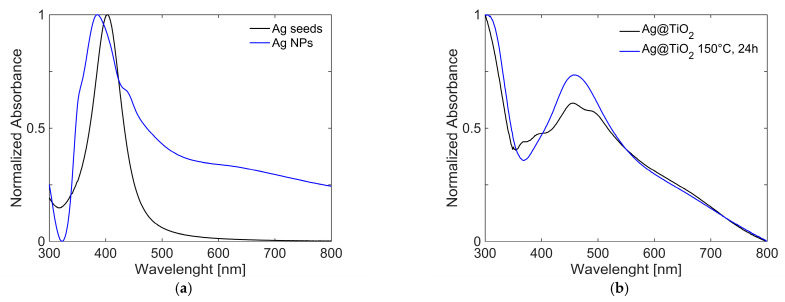
UV/Vis spectra of products at different steps of the synthesis: (**a**) silver seeds (black line) and Ag Nps (blue line) and (**b**) Ag@TiO_2_ in ethanol, as synthetized (black line) and after 24 h (blue line).

After the growth step, Ag NPs showed a more complex absorption profile, as reported in Figure 7a. This can be attributed to the formation of particles with different morphologies and, therefore, different sizes. The peak originally centred at 430 nm became a shoulder, whereas additional absorption bands emerged. These are associated with the presence of nanocubes [53], observed at ~350 and 380 nm, and nanorods [54], which contribute to the features at ~380 and 620 nm (as will be confirmed by TEM data shown below).

The growth of the coating with TiO_2_ is confirmed by the red shift of the plasmonic absorption bands, observed in Figure 7b, caused by the increase in the local refractive index around the Ag NPs due to the surrounding medium changes from water to titania and the high extinction at low wavelength (<350 nm). Solvothermal treatment, carried out at 150 °C for 24 h, appears to slightly modify the morphology of the material, as evidenced by changes in the absorption spectra. The final nanocomposites present great stability over time, which was checked with UV-Visible spectra (reported in SI, Figure S3).

**Figure 8:**
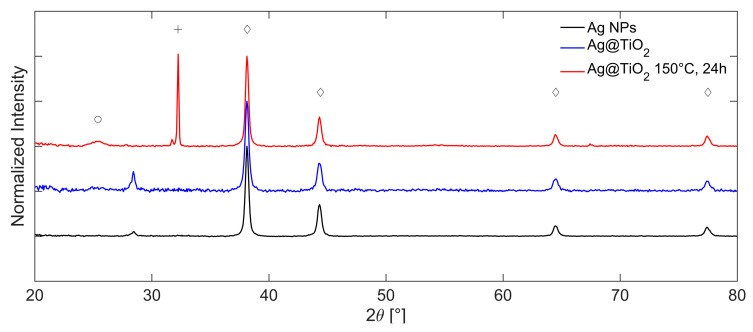
Comparison between the XRD diffractograms, acquired in BB configuration, of Ag NPs (black) and Ag@TiO_2_ in ethanol before (blue) and after (red) solvothermal treatment at 150 °C for 24 h, with the assignation of Ag-O (+), anatase (○), and silver (◇) diffraction peaks.

The corresponding XRD diffractograms collected in a Bragg Brentano (BB) configuration at each step of the synthesis are reported in Figure 8, where the intensity has been normalized with respect to the most intense reflection of cubic Ag at 2θ = 38.10°. The characteristic diffraction pattern of cubic silver is colored in black and marked with diamonds. It displays reflections at 2θ = 38.10, 44.27, 64.43, and 77.37°, corresponding to the crystallographic planes (111), (200), (220), and (311), respectively (ICDD 01-087-0597). Furthermore, a weak reflection attributed to titanium dioxide in the anatase phase appears at approximately 2θ = 25.43° (marked with a circle, red pattern, ICDD 00-004-0477). The low intensity of this peak suggests a limited degree of crystallinity, likely resulting from the reflux-based sol–gel process. Two additional signals at 2θ = 28.42° and 32.21° are associated with the zero-background silica support and with the presence of silver oxide (Ag-O, ICDD 01-076-1489) [55], respectively. The comparison between the theoretical lattice spacings and the experimental values obtained by the analysis of the diffraction pattern is shown in SI, Table S3.

After solvothermal treatment at 150 °C for 24 h, the anatase peak (red line) becomes slightly more intense, indicating an increased degree of crystallinity due to enhanced thermal conditions.

Crystallite sizes are estimated from reflection broadening via Scherrer analysis, yielding values of approximately 25 nm for silver and 10 nm for TiO_2_. The average crystallite size of the silver domains remains essentially unchanged compared to the pretreatment condition, indicating that the solvothermal treatment did not affect Ag NPs. 

**Figure 9:**
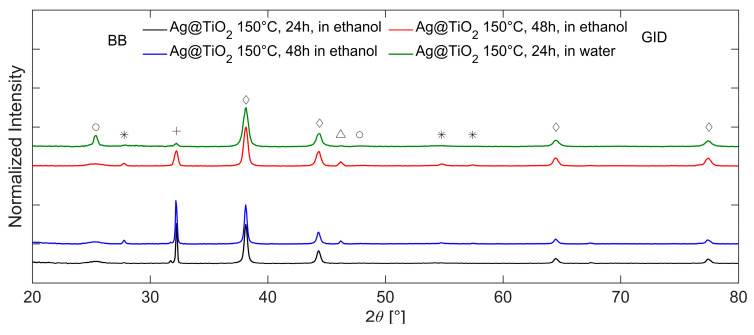
XRD diffractograms of Ag@TiO_2_ after solvothermal treatment at 150 °C in ethanol for 24 h (black line) and 48 h (blue line), acquired in BB configuration. XRD diffractograms, acquired in GID configuration of Ag@TiO_2_ after solvothermal treatment at 150 °C in ethanol for 48 h (red line) and in water for 24 h (green line). The reflection assignments are anatase (○), rutile (∗), Ag-O (+), cubic Ag (◇), and Na_2_O (Δ).

The influence of the solvothermal treatment time (24–48 h) and the solvent composition (H_2_O–EtOH) used in the liner vessel on the synthetized NPs is shown in Figure 9, respectively. It is important to note that these measurements were performed using Grazing Incidence Diffraction (GID) geometry and BB geometry, and the intensity of diffractograms is normalized with respect to the most intense reflection of cubic Ag.

Extending the treatment time of the sample in ethanol from 24 (black line) to 48 h (blue line) leads to a slight increase in the crystallinity of titania. However, it also causes significant changes in the overall diffractogram. Specifically, new reflections attributed to the rutile phase of TiO_2_ appear at 2θ = 27.76°, 54.74°, and 57.40° (ICDD 01-086-01147); the peaks are marked with a star. These reflections are sharper than those of anatase, indicating higher crystallinity of the rutile phase. The size of the anatase crystallites calculated with respect to the most intense reflection at 25.26° is approximately 7 nm, while the size of the rutile crystallites calculated with respect to the reflection at 27.77° is approximately 50 nm. The intensity of the Ag-O phases’ [55] reflection at 2θ = 32.18° is comparable to that of the sample treated for 24 h. Furthermore, a new reflection at 2θ = 46.19° appears; this is attributed to Na_2_O (ICDD 00-023-0528), deriving from the residual of sodium citrate used for Ag synthesis.

The variation of the solvent in the bomb vessel from ethanol to water leads to a significant increase in the crystallinity of titania in the anatase phase. This is evidenced by the sharper diffraction signal at 2θ = 25.32° (green line), acquired in the GID configuration, compared to the sample treated in ethanol for 48 h (red line). By comparing the spectra, it is possible to observe not only the increase in reflection intensity but also the reduction in reflection width, which leads to the estimate of a change in crystallite size from 7 to 165 nm. The solvothermal treatment induces the preferential growth of plane (101), which has already been observed in the literature [56,57].

**Figure 10:**
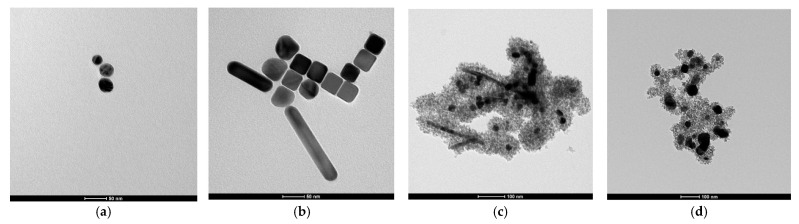
TEM images of each reaction step product: (**a**) Ag seeds; (**b**) Ag NPs; (**c**) Ag@TiO_2_ nanocomposite; (**d**) Ag@TiO_2_ nanocomposite in ethanol treated at 150 °C for 24 h. It is helpful to point out that the scale bar of (**a**) and (**b**) is 50 nm, while that of (**c**) and (**d**) is 100 nm.

Investigation of the size and morphology of the synthesized materials was performed using TEM. The image of the seed suspension, reported in Figure 10a, confirms the size estimate obtained from UV/Vis spectroscopy: the nanoparticles are spherical, with an average diameter of 26 ± 9 nm (the error corresponds to the standard deviation of the measured data). Similarly, for the Ag core grown, the UV/Vis predictions are corroborated by TEM analysis, as observed in Figure 10b. The images reveal nanoparticles with a variety of morphologies, resulting from a not fully controlled growth process. Regarding seed NPs, the particle size was determined by measuring the diameter of individual particles; however, in this case, only those with an aspect ratio (AR) < 1.3 were considered to exclude nanorods from the analysis. This selection yielded an average diameter of 46 ± 9 nm. The two distribution graphs for Ag seeds and NPs are reported in the SI, Figure S4a,b, respectively. To ensure completeness, TEM images of different samples are presented in Figure S5 of the SI.

In the case of TiO_2_ coverage, the TEM image of Figure 10c shows the quality of shell formation, always surrounding the metal cores, without the presence of titania agglomerates alone. Finally, solvothermal treatment, whose output is reported in Figure 10d, leads to a moderate agglomeration and size growth.

**Figure 11:**
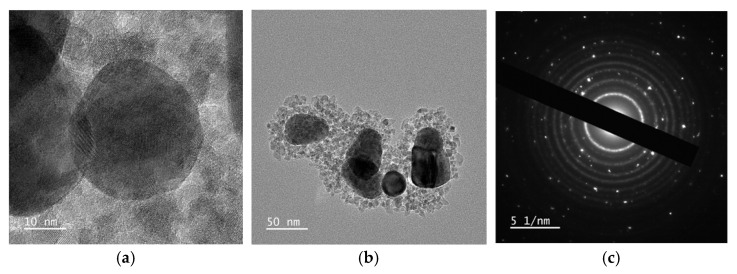
TEM images, acquired in diffraction mode, of each reaction step product: (**a**) Ag seeds, (**b**) Ag NPs, and (**c**) Ag@TiO_2_ nanocomposite in ethanol in reciprocal space.

HR-TEM was also used in the diffraction mode. In Figure 11c, the diffraction pattern corresponding to the image in Figure 11b is analyzed. Bright spots, associated with the crystalline planes of silver, are clearly visible, along with concentric rings corresponding to crystallized titania nanoparticles. The assignment is based on the fact that silver, due to its limited number of crystallographic orientations, produces discrete diffraction spots, whereas the large number of differently oriented TiO_2_ nanoparticles results in continuous diffraction across all directions. This leads to the formation of complete rings, as each individual spot merges into a circular pattern. Each pair of symmetric spots with respect to the central bright region corresponds to a specific interplanar spacing d of the crystal lattice. This spacing can be calculated using the equation *d* [nm] = 1/(*x*/2), where *x* is the distance between the symmetric diffraction points, measured in nm^−1^. All the calculated distances are compared in Table S3 of the SI with literature values. Figure 11c represents the reciprocal space image of the Ag@TiO_2_ nanocomposite.

**Figure 12:**
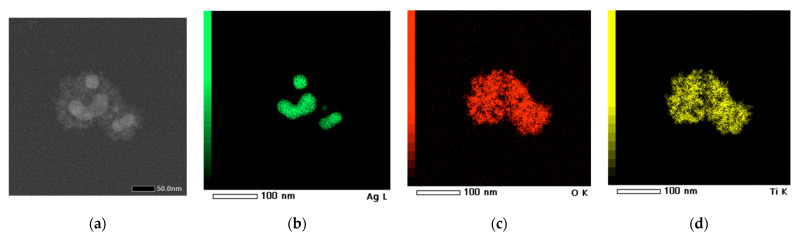
Ag@TiO_2_ in ethanol for 24 h (**a**) TEM images; (**b**) Ag, (**c**) O, and (**d**) Ti elemental mapping.

EDS analysis and STEM were employed to gain a comprehensive understanding of both the morphology and composition of the synthesized material. The quality of the TiO_2_ coating after solvothermal treatment, which applies considerable stress due to subcritical reaction conditions, was confirmed through the elemental mapping of the nanocomposite clusters. Figure 12a shows the region of nanosystems investigated, while Figure 12b–d display the elemental distribution of Ag, O, and Ti, respectively, confirming the successful formation of a core–shell-like structure. Additionally, an EDS spectrum was created to quantitatively assess the elemental composition (Table 1), verifying that the Ti:O ratio remains close to the stoichiometric value of 1:2.

**Figure 13:**
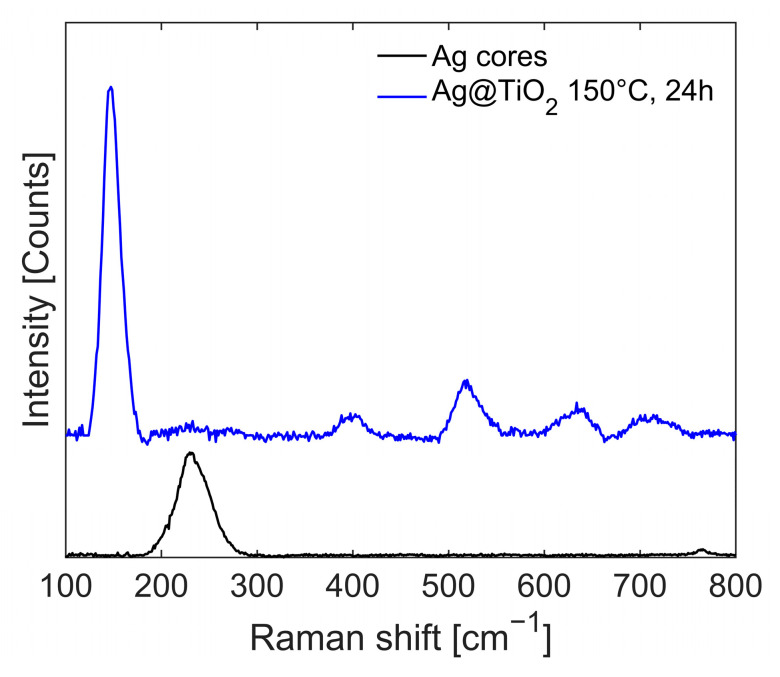
Raman spectra of the reaction products: Ag NPs (black line) and Ag@TiO_2_ after solvothermal treatment (blue line).

The Raman spectra of the reaction product at each synthesis step are reported in Figure 13. Ag NPs (black line) shows an intense peak at ν = 241 cm^−1^, related to the normal Ag–O vibrational mode. This signal disappears after the sol-gel reaction, and the initially amorphous titania structure does not exhibit any significant peaks (not reported in the figure). However, after solvothermal treatment (150 °C, 24 h, in water), the characteristic peaks of anatase, $\tilde{\upsilon }=148,399,516,635cm^{-1}$, dominate the spectrum (blue line).

In order to proceed with the determination of the nanothermometry properties, a scan at different input powers was performed on samples to verify in which power to work in order to avoid the heating effect of the laser.

**Figure 14:**
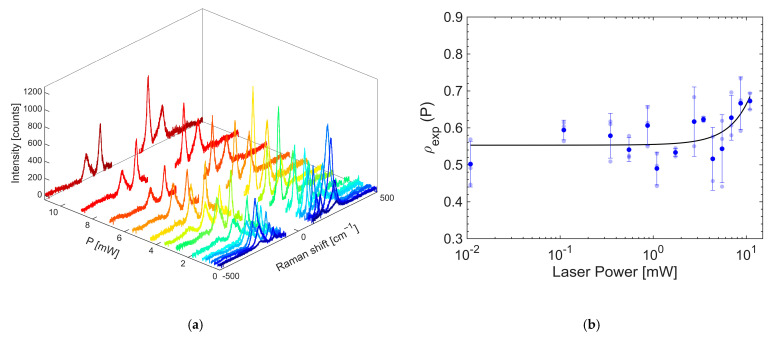
(**a**) Raman spectra at increasing powers of OP4 at 800 nm. (**b**) Thermometer indicator values versus power of the excitation laser. The line is a polynomial interpolation of data serving as an eye guide for the behavior.

The Raman Stokes and anti-Stokes spectra recorded at a given power are shown in Figure 14a, while in (b) the values of the ratios of the anti-Stokes to Stokes intensities (used as the thermometric indicator ρ) are shown as a function of the input power on the logarithm scale. The appreciable width of the error bars reported at the points is caused by the inhomogeneity of the sample. Via polynomial interpolation of the ρ values, it was found that up to 3 mW power, there is a constant relationship between the thermometric indicator and the power used. Above that, however, there is exponential-like growth due to the heating of the sample by the laser. The same trend in power was observed for the other excitation wavelengths employed. We therefore proceeded to work in the linearity regime, keeping the laser below 3 mW power and trying to keep it as similar as possible for all samples at the wavelengths employed.

**Figure 15:**
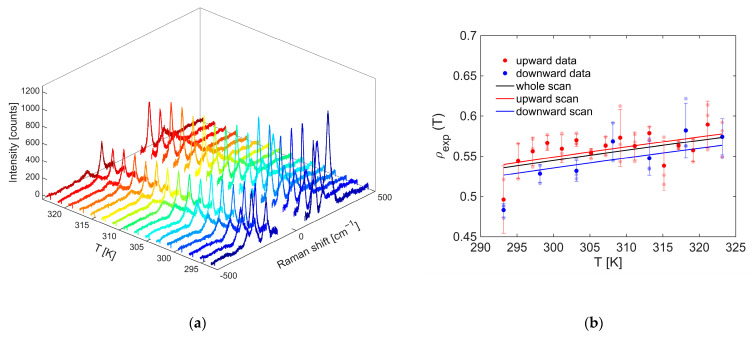
(**a**) Raman spectra recorded at increasing temperatures. (**b**) Anti-Stokes/Stokes intensity ratio as a function of temperature. The data were collected in the temperature range from 20 to 50 °C, at 800 nm, with input power of 1.0 mW.

The nanothermometry measurement recorded for the OP4 synthesis sample by exciting at 800 nm with an input power of 1.0 mW is shown in Figure 15a.

The relationship between the intensity ratio of the anti-Stokes and Stokes signals, collected as the thermostat temperature increases, is represented by the red dots in Figure 15b. On the other hand, those in blue refer to a descending scan in which the temperature was decreased every 5 °C. At each temperature set by the thermostat, three pairs of Stokes and anti-Stokes spectra were recorded, from which three ρ values of the thermometer indicator were calculated, respectively, represented by the dots with a faint red or blue coloration. From the average of the three ratios obtained at a given temperature, the values of the most frequently marked points were obtained, with which the respective error bars are associated. It can be seen that the errors found are comparable with those of nanothermometries performed in previous works [19,20,21]. Moreover, all slopes were positive, as expected, due to the fact that the population of the excited vibrational level from which the transition into anti-Stokes is observed at −143 cm^−1^ increases as the temperature increases according to the Boltzmann statistic. Additionally, as the number of points considered to calculate the global slope increased, the relative sensitivity improved.

The summary of the nanothermometry measurements performed on OP4 and TiO_2_ samples, with different excitation lengths λ_exc_ in the visible range (at 530 nm) [58] or in the NIR (at 800, 850, and 900 nm) is reported in Table 2. Working at 530 nm, a value very similar to that of nanothermometries at 800 nm, i.e., 0.26 K^−1^%, is obtained. S_rel_ results are almost constant by moving to the 850 and 900 nm excitation wavelengths. The relative sensitivity obtained with nanocomposites is comparable to those obtained in the literature on commercial anatase [19,20].

To understand whether the presence of the silver core leads to an improved performance of the nanothermometer, it is possible to compare the relative sensitivity values. Data reported in Table 2 show that Ag@TiO_2_ possess sensitivity values slightly higher than those of anatase in the visible range. The nanocomposite seems to lead to increased sensitivity in comparison to anatase nanoparticles. It is expected that the possibility of obtaining a homogeneous powder of this nanomaterial will probably allow for further improvement in its nanothermometric properties to be observed. What can be safely stated at present is that the anatase incorporated in the synthesized nanocomposite can be used as a Raman nanothermometer to determine the local temperature.

**Figure 16:**
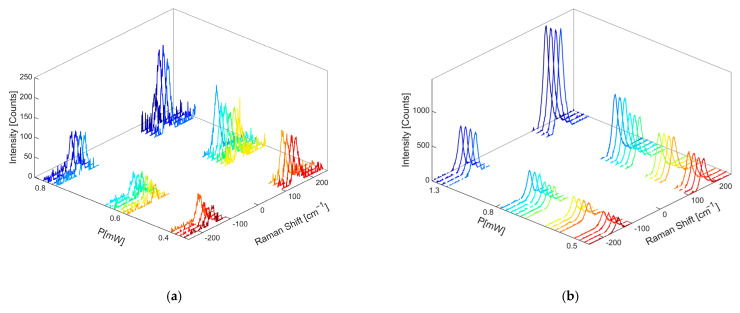

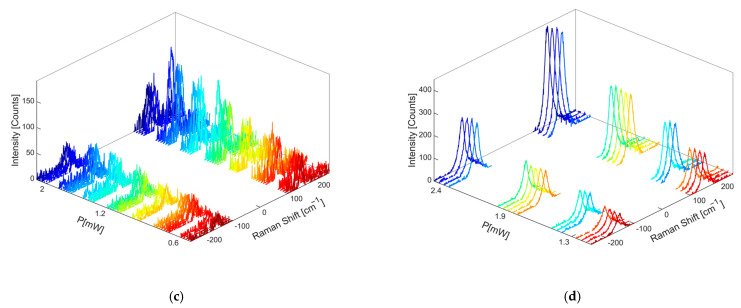
Raman spectra of two-steps Ag@TiO_2_ sample in water, measured at (**a**) 530 nm and (**c**) 633 nm, and anatase NPs, measured at (**b**) 530 nm and (**d**) 633 nm, at different input powers.

Using Raman measurements made at the various powers of the incident laser of the OP4 sample, reported in Figure 14, and the Ag@TiO_2_ sample in water, reported in Figure 16, it was also possible to assess the extent of local heating induced through laser excitation. As an example, OP4 shows a temperature rise of a few tens of degrees as the power incident on the sample increases in the visible and near-IR range. The behavior of Ag@TiO_2_ sample in water at 530 nm is comparable. Moreover, it can be observed that the thermal release of the nanocomposites at the wavelength of 530 nm is higher than that of anatase NPs, while at the wavelength of 633 nm, it is lower. This is related to the fact that at 530 nm, the excitation is in partial resonance with the silver plasmonic band, while at 633 nm, it is completely out of resonance. The evidence of an increase in temperature of anatase NPs in the near-IR range has already been reported in the literature [20].

## 3. Conclusions

In this work, Ag@TiO_2_ nanocomposite nanoparticles were proposed as photothermal agents; the anatase nanothermometer is arranged around the silver nanoparticles, which are used as nanoheaters. By exciting Ag NPs under plasmonic resonance conditions, it is indeed possible to cause the localized release of large amounts of heat to realize hyperthermia conditions.

To create the nanocomposites, two bottom-up sol-gel methodologies were designed and tested: starting with one-pot synthesis, a two-step synthesis method was developed, in which focus was paid to choosing reagents and reactions that are as environmentally friendly and green as possible.

The purpose of the one-pot method was to obtain crystalline Ag@TiO_2_ nanocomposite nanoparticles by their formation within the same reaction environment, followed by solvothermal treatment for conversion to crystalline anatase. From the precursor suspension of Ag NPs and anatase shell, the respective nanoparticles were also synthesized for use as a comparison.

The formation of the amorphous shell for OP syntheses was confirmed via UV/Vis by the redshift of the plasmon of about 10 nm compared with that of the uncoated core, which was due to the high refractive index of titania. The coating process was successfully monitored using UV/Vis methods, where the intensity growth of the plasmonic band was observed over time, even at the end of the reaction.

The Raman signals of the anatase shell and silver core were clearly observed in OP4 and OP8 samples, where the color change of the suspension from black to sand-brown after crystallization, induced with a solvothermal treatment, also occurred. For OP4, High-Resolution TEM coupled with EDS analysis established the formation of a diffuse anatase layer around Ag NPs with certainty. A critical factor in one-pot synthesis, however, lies in the polydispersity observed in the synthetized samples, which could have important implications for its final applications.

The two-step synthesis method was developed to pursue a sort of control over the core size, the reproducibility of the synthesis, and to eliminate DMF from the reaction as a hazardous and non-green solvent.

The phase transformation of the oxide, from amorphous to crystalline, was accomplished through solvothermal treatment. Different experimental parameters, such as time and solvents, were tested to obtain the best-performing process in terms of the degree of crystallinity of the oxide. The presence of the silver core and anatase was verified in all samples created.

The two-step process is advantageous in that it enables the replacement of the non-green reducing agent DMF with citrate, working in an ethanol/aqueous solution, and the removal of the purification stage at the end of shell formation during one-pot synthesis. In this way, a narrow size distribution of the silver nanoparticles that will form the core, centered from 10 to 30 nm in diameter, was obtained, and the coating and shell crystallization stage occurred in a single stage, with solvothermal treatments outperforming with respect to the one-pot method. Thus, two-step synthesis would seem promising to obtain small-sized coated cores capable of penetrating the cell membrane for photothermal therapy (<60 nm) [12].

A critical issue lies in the control of the dispersion of the realized nanostructures. One of the parameters that needs to be better controlled in the future is the amount of titanium oxide that induces the formation of a thick layer, leading to the formation of large clusters consisting of the assembly of multiple Ag NPs embedded in a TiO_2_ shell. Trials at different concentrations of Ti precursor could help find ideal conditions for controlling the coating thickness of individual Ag NPs to achieve a greater dispersion of individual nanostructures.

Nanothermometry measurements conducted on nanocomposites and anatase samples, using various excitation wavelengths in the visible range, at 530 nm, and in the near-infrared, at 800, 850, and 900 nm, show a relative sensitivity in the range of 0.24 to 0.26 K^−1^%. It should be noted that the relative sensitivity achieved with these nanocomposites is comparable, or even slightly better, to those reported in the literature for commercial anatase. This comparison provides context for the performance of the samples studied within the broader field of nanothermometry.

A comparison of relative sensitivity values is a key method to evaluate whether the silver core in Ag@TiO_2_ nanocomposites improves the performance of the nanothermometer. This analysis reveals that Ag@TiO_2_ nanocomposites demonstrate slightly higher relative sensitivity values compared to pure anatase TiO_2_ nanoparticles. This observation suggests that the nanocomposite structure indeed leads to an improvement in the sensitivity for temperature measurements.

Although the current results are promising, there is potential for further improvement. The development of a method to produce a more homogeneous powder of this nanomaterial could lead to even better nanothermometric properties. This homogeneity could ensure more consistent and reliable temperature measurements across the sample.

It is important to note that while further optimizations are possible, the current findings already demonstrate a significant achievement: the anatase TiO_2_ component in the synthesized Ag@TiO_2_ nanocomposite is effective as a Raman nanothermometer for local temperature determination.

In conclusion, the Ag@TiO_2_ nanocomposite shows enhanced sensitivity compared to pure anatase, making it a promising candidate for nanoscale temperature-sensing applications.

## 4. Materials and Methods

Nanocomposite nanoparticles consisting of an Ag core covered by TiO_2_ were suitably synthesized through a one-pot method and a two-step process, followed by solvothermal treatment. They were then characterized by UV/Vis spectroscopy, X-Ray Diffraction, High Resolution Transmission Electronic Microscopy, and Raman spectroscopy.

### 4.1. Synthesis

#### 4.1.1. Chemicals

Silver nitrate (AgNO_3_), cetyltrimethylammonium chloride solution (CTAC, C_19_H_42_ClN), sodium borohydride (NaBH_4_), acetylacetone (C_5_H_8_O_2_), and ascorbic acid (C_6_H_8_O_6_) were purchased from Sigma-Aldrich (Sigma-Aldrich, Merck, Milano, Italy). Tri-sodium citrate dihydrate (Na_3_C_6_H_5_O_7_·2H_2_O) and titanium(IV) butoxide (C_16_H_36_O_4_Ti) were obtained from Fluka (Honeywell, Charlotte, NC, USA). Ethanol (C_2_H_6_O) was provided by Carlo Erba (Carlo Erba, Milano, Italy), and N,N-dimethylformamide (DMF, C_3_H_7_NO) by Merck (Merck, Milano, Italy). Sodium sulfide (Na_2_S) was purchased from Riedel-de Haën (Honeywell, Charlotte, NC, USA).

#### 4.1.2. Silver Nanoparticles

For the synthesis of silver nanoparticles in aqueous solution, the literature procedure outlined by Lee et al. [59] was adapted. A solution of silver nitrate in water was prepared in a suitable flask, while a 0.01 mg/mL solution of sodium citrate dihydrate in water was prepared in a 5 mL glass vial. The first was poured into a three-necked flask and, by heating with silicone oil on a hot plate and magnetic stirring, was refluxed. Then, the second solution was added, and the mixture gradually turned yellow-red within 20 min, then gray-green within the next 30 min. The contents of the flask were collected in a 250 mL glass bottle and allowed to cool at room temperature. Centrifugation was then carried out, washing twice with water. After vacuum drying, a black solid with metallic highlights was obtained.

The specific amounts used in the syntheses of Ag NPs are 22 mg of AgNO_3_, 27 mg of sodium citrate dihydrate, 100 mL of H_2_O, and a reflux time of 70 min.

#### 4.1.3. Anatase Nanoparticles

The synthesis of TiO_2_ NPs was adapted from the one-pot core–shell synthesis of Ag@TiO_2_ outlined by Liz-Marzán et al. [23]. A 5.75 mM TOB/acetylacetone solution in ethanol was prepared in a 100 mL flask. This was transferred to a 250 mL three-neck flask and refluxed by heating with a silicone bath on a hot plate and with magnetic stirring. Water was added until an opaque white coloration was observed to develop (4 mL total), then allowed to react for 90 min. The suspension was allowed to cool in a 100 mL glass bottle, then centrifuged and washed 2 times with ethanol. After transfer to a 50 mL falcon, it was placed in an oven in a 150 mL Teflon liner at 150 °C for 24 h using a filling ratio of 50%. At the end of the solvothermal treatment, the precipitate at the bottom was dissolved in ethanol, resulting in a milky white suspension. After centrifugation and two washes with ethanol, the white solid was allowed to dry under vacuum. A brown sample was obtained which, after grinding for Raman and XRD analysis, became white.

#### 4.1.4. One-Pot

**Figure 17:**
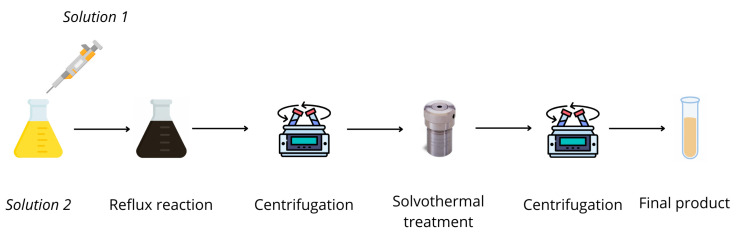
Reaction scheme for one-pot syntheses followed by solvothermal treatment.

For nanocomposite synthesis of Ag@TiO_2_, the one-pot procedure outlined by Liz-Marzán et al. [23] was modified. The procedure is shown schematically in Figure 17. A solution of TOB/acetylacetone 5.75 mM in ethanol was prepared in a 100 mL flask, mixed and sonicated before use (*solution 1*). In a 50 mL falcon protected by aluminum foil, a solution of 3.8 mM silver nitrate and 0.8 M water in 30 mL DMF was prepared (*solution 2*). Upon addition of the reducing agent, the solution took on a progressively increasing yellow coloration. Two aliquots of *solutions 1* and *2* were combined in a 250 mL three-neck flask in a volumetric ratio of 4:1.

The reactions were performed at molar ratios of titania to silver ranging from 5.0:1 to 6.2:1, obtained by varying the quantity of AgNO_3_ (in the range of 21–23 mg), and the volume of *solution 1* (from 20 to 100 mL) and *solution 2* (from 5 to 25 mL). Details on the quantities for reactions can be found in Table S1 of SI. Using silicone oil bath on a hot plate and with magnetic stirring, the mixture was refluxed, for different times, from 80 to 120 min. The reaction was allowed to proceed for at least 80 min, during which it first turned pinkish, then brownish-red, and finally black-brown. After refluxing was completed, the suspension was transferred to a 100 mL glass bottle and allowed to cool. To remove the DMF present, it was centrifuged on Teflon tubes and washed twice with ethanol. The black precipitate was brought into suspension in ethanol on a 50 mL falcon, then transferred to a Teflon liner and left in an oven at 150 °C for 24 h. The proceeds were centrifuged and washed twice with ethanol. The final solid was left to dry under vacuum before analysis. Images of sample OP4 are reported in SI (Figure S1) as example.

#### 4.1.5. Two-Steps

**Figure 18:**
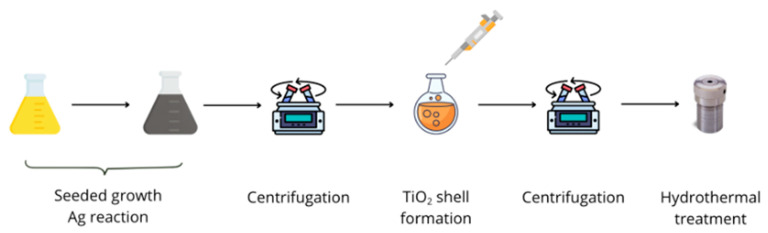
General scheme of two-steps Ag@TiO_2_ synthesis.

The two-steps synthesis involves the preparation of silver seeds that are grown and later covered with an amorphous TiO_2_ layer subjected to a solvothermal crystallization treatment. The scheme of the process is shown in Figure 18.

##### Synthesis of Ag NPs Core

The synthesis of Ag NPs is based on a seeded-growth method, and the work of Hong et al. [44] was used as starting point.

The seeds (AS) were formed via mixing in a 30 mL flask: 10 mL of ultrapure water, 40 μL of CTAC (0.125 M), 25 μL of AgNO_3_ (0.01 M), and 450 μL of NaBH_4_ (0.01 M). The solution was maintained at T = 30 °C for a period of 40 min under vigorous stirring. It is of paramount importance to emphasize that subsequent syntheses were invariably carried out in the same order of reagent addition to ensure the optimal reproducibility of the synthetic method. It is evident that the reduction of silver is effective, based on the observation of an immediate change in the color of the suspension to yellow. Subsequently, 10 mL of the as prepared AS was transferred into a 100 mL round-bottom flask containing 10 mL of AgNO_3_ (0.1 M), 40 mL of CTAC (30 mM), and 0.5 mL of Na_2_S (10 mM); also, during this step, the order of the reagents was invariable. The suspension was kept at T = 70 °C under vigorous stirring for 20 min. Thereafter, 15 mL of ascorbic acid (0.50 M) was added to the mixture, and the reaction was maintained under the same conditions for a further 60 min. The product of the reaction was then cooled at room temperature, and the Ag NPs were purified by two centrifugation steps (4650 RCF, 20 min each). Following this, the Ag NPs were then redispersed in 20 mL of water.

##### Synthesis of Titania Shell

The coverage of silver nanoparticles was performed by mixing 45 μL of TOB with 30 mL of ethanol with magnetic stirring for 15 min to induce homogenization of the suspension. Then, 5 mL of Ag NPs suspension was added, and after 20 min of stirring at RT, two centrifuges were performed (7690 RCF, 5 min each), and the final pellets were dispersed in 30 mL of water.

The obtained suspension was heated to 100 °C and stirred for a period of 2 h. The final step is known to induce the hydrolysis of butoxide and the formation of a coverage gel shell. The reactions involved in the sol-gel process are reported in Table 3. The reaction product was centrifuged twice (7690 RCF, 5 min each), and the final pellet was dispersed in 30 mL of ethanol, water, or a mixture of both.

##### Crystallization of Titania

The final synthesis step is based on a solvothermal process to induce growth in the anatase crystal dimension. In this work, a 23 mL Teflon liner vessel filled with 5 mL of suspension was used for the treatment. The filling ratio $\left(\frac{volume\;of\;suspension\;\left[ml\right]}{volume\;of\;the\;liner\;\left[ml\right]}\right)$ was a critical parameter because of the effect of liquid vapour equilibrium on the inner conditions [60]. Experimental parameters were tested to reach optimal conditions for anatase phase formation: the time in the oven (24–48 h) and the solvent ($\frac{volume\;H_{2}O}{volume\;EtOH}=\infty ,\;3,\;0.33,\;0)$. The overview of samples is reported in SI, Table S2.

### 4.2. Characterization Techniques

#### 4.2.1. Absorption

Spectra were recorded using an Agilent Cary 5000 UV-Vis-NIR dual-beam spectrophotometer (Agilent, Santa Clara, CA, USA) employing two 10 × 10 mm optical path quartz fluorescence cuvettes in the Cary WinUV Scan program (Agilent, Santa Clara, CA, USA). Before each set of measurements, the baseline was recorded to remove the solvent contribution from them and obtain the balance of the two measurement beams.

#### 4.2.2. XRD

XRD diffractograms were recorded using two different diffractometers: a D8 Advance Plus diffractometer (Bruker, Billerica, MA, USA) in Bragg–Brentano geometry using the Kα radiation of copper (λ = 1.5406 Å) as the source, and a Panalytical Empyrean diffractometer (Malvern Panalytical, ad Almelo, The Netherland) equipped with a Cu X-ray tube and PPC (parallel plate collimator) detector operating in grazing-incidence mode (W incidence angle = 1°). The radiation used in the second diffractometer was Cu Kα (l = 1.54056 Å), operating at 40 kV and 40 mA. All patterns were collected in the 20° ÷ 80° 2q range.

The vacuum-dried samples were ground in an agate mortar before being transferred to a silicon zero background sample holder. Suspensions in ethanol, on the other hand, were suitably deposited drop by drop on slides by removing the solvent via hot plate.

Processing of the collected data was aimed at recognizing characteristic reflections and establishing the percentage abundance of anatase and silver. Using the program DIFFRAC.EVA, Bruker (Bruker, Billerica, MA, USA), the crystalline phases of the samples were identified. Using the XRD pattern, the crystallites size was calculated through Scherrer’s Equation $D=\frac{k\cdot \lambda }{\cos\theta \cdot \sqrt{{FWHM}^{2}-B^{2}}}$, where k is the coefficient of the particles’ shape (dimensionless shape factor with a value close to unity, where we used 0.9); λ is the X-ray radiation wavelength; θ is Bragg’s angle; *FWMH* (Full Width at Half Maximum) is the line broadening at half the maximum intensity (in radians); and *B* = instrumental line broadening (in radians), measured using Lanthanum hexaboride (LaB6) as standard.

#### 4.2.3. TEM

HRTEM analysis was performed by depositing a few drops of suspension on the grid of an FEI Tecnai G2 TEM. Solid samples of one-pot synthesis solubilized in ethanol were investigated using a JEOL JEM-2100F microscope (UHR) (Jeol, Madrid, Spain) coupled with an Oxford UltimMax energy-dispersive X-ray spectroscopy (EDS) (Oxford instrument Overseas España, Madrid, Spain) detector at the CIC BiomaGUNE laboratory, San Sebastián. Using EDS technique, chemical information about the sample was obtained [61]. The spatial resolution ranged from 10 nm to a few μm.

The two-step samples were observed using a JEOL F200 Transmission Electron Microscope (Jeol Italia, Milano, Italy) equipped with a cold field emission gun. The microstructure was characterized via either high-resolution imaging in TEM mode (HRTEM) (Jeol Italia, Milano, Italy), diffraction, o high-angle annular dark-field scanning transmission electron microscopy (HAADF-STEM) (Jeol Italia, Milano, Italy). High-resolution images of the morphology of a specimen were investigated using the secondary electrons detector operating in STEM mode. Elemental analysis and mapping were performed using a JEOL 100 mm^2^ silicon drift energy-dispersive X-ray spectrometer (EDS). The samples were prepared by drop-casting the dispersions on a carbon-supported copper grid (400 mesh size).

#### 4.2.4. Raman

Two different Raman set ups were used to record Raman spectra on one-pot and two-step samples.

The first Raman apparatus employs an Ar^+^ (Spectra Physics) laser, operating at 514 nm. It is coupled with a microscope (Olympus BX 40, Tokyo, Japan) equipped with 4×, 10×, 20×, 50×, and 100× objectives (Olympus SLMPL, Tokyo, Japan). Appropriate edge and notch filters are present to select Raman Stokes transitions and remove the contribution of elastic scattering from the spectrum, respectively. Scattered radiation passes through the spectrograph (Triax-320, ISA Instruments, Jobin Yvon, Roma, Italy) and is detected using a liquid nitrogen-cooled CCD photon-counting system (Horiba Scientific Symphony II FIOE, Rome, Italy).

**Figure 19:**
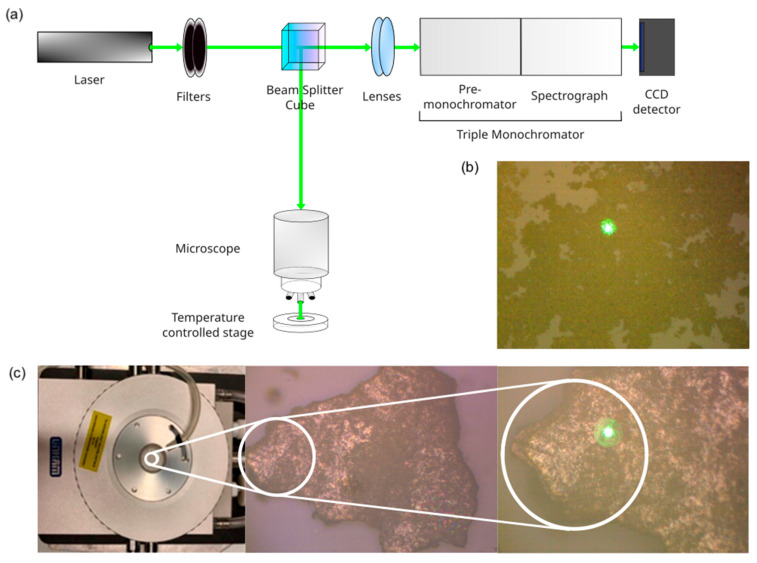
(**a**) Image and scheme of the micro-Raman of the second set-up (Triple Raman). (**b**) Image with laser spot incident on the sample Ag@TiO_2_ in water. (**c**) Thermostat employed with magnification on the laser spot incident on the sample OP4 inside the thermostat.

The second one is the Triple Raman apparatus, shown in Figure 19. It is equipped with a Ti:Sapphire continuous wave (CW) laser, modulated in the 675–1000 nm range (MKS Instruments, Spectra Physics, 3900S, Santa Clara, CA, USA), pumped by a CW Optical Pumped Semiconductor Laser (Coherent, Verdi G7, Santa Clara, CA, USA) and a He-Ne CW laser, operating at 633 nm (Model 05 LHP 991, Melles Griot, Carlsbad, CA, USA). The laser is coupled with a microscope (Olympus BX 40, Tokyo, Japan) and focused on the sample with 4× and 20× objectives (Olympus SLMPL, Tokyo, Japan). The scattered radiation from the sample is collected in the slits of a triple spectrograph (Jobin Yvon S3000, Horiba, Kyoto, Japan) consisting of a double monochromator (Jobin Yvon, DHR 320, Horiba, Kyoto, Japan) with a modulable filter to remove Rayleigh radiation and an actual spectrograph (Jobin Yvon, HR 640, Horiba, Kyoto, Japan). Detection is by a liquid nitrogen-cooled CCD (Jobin Yvon, Symphony 1024 × 256 pixels). Prior to each measurement, a calibration was performed by measuring the spectra of a silicon slab (${\tilde{\nu }}_{max}=520\;cm^{-1})$ and/or a vial containing cyclohexane (${\tilde{\nu }}_{max}=800\;cm^{-1})$. Initial processing of the recorded spectra was performed by subtracting the baseline when a background signal was present. Then, the intensity of the remaining signals was determined using Lorentzian interpolation.

### 4.3. Nanothermometry Protocol

Nanothermometry measurements were made with Triple Raman by employing a thermostat cell (Linkam, THMS600/720, Tadworth, UK) to control and vary the temperature of the sample placed in it. Heating and cooling took place at a rate of 5 K/min and were provided by acting on the electrical resistance inside the thermostat and on a flow of liquid nitrogen from an external reservoir, respectively. The latter was also used to remove the air inside the cell and create an inert atmosphere whenever a new sample was placed in the thermostat. For the set temperature to be reached and stabilized, it was decided to wait at least 15 min. Thus, a sensitivity of 0.1 K can be achieved by varying the temperature in the range of −196 to 600 °C. For the nanothermometers, Stokes and anti-Stokes spectra of the sample were recorded by repeating the measurement three times for each excitation wavelength (530, 800, 850, and 900 nm), set temperature (20 to 50 °C), and laser power used on the sample modulated by acting on the Optical Density (OD) filters. The Raman Stokes and anti-Stokes spectra were collected at temperatures ranging from 20 to 50 °C and recorded every 2 °C as the temperature increased (or decreased). The data were collected with HORIBA’s LabSpec software (Horiba Italia, Torino, Italy) and then processed using an appropriate Matlab program (Matlab, Milano, Italy).

## Figures and Tables

**Table 1:** Elemental abundance (atomic percentage) and energy of the most intense peak in the EDS spectra, reported in Figure 12, for O, Ag, and Ti.

Element	%Atom
O	54.81
Ag	21.65
Ti	23.54

**Table 2:** Overview of nanothermometry measurements on samples of OP4 and TiO_2_ anatase NPs.

Sample	Excitation Wavelength [nm]	Laser Power [mW]	Raman Peak [cm^−1^]	Relative Sensitivity @ 295.15 K [K^−1^%]
Ag@TiO_2_ [58]	530	1.4	148.6	0.26
TiO_2_ [58]	530	1.5	143.8	0.24
Ag@TiO_2_	800	1.0	149.1	0.24
	850	1.6	149.4	0.25
	900	2.8	149.7	0.25
TiO_2_	800	1.6	146.1	0.24

**Table 3:** Reactions involved in the sol-gel process.

Reaction	Mechanism
Hydrolysis	Ti(OBu)_4_ + H_2_O → Ti(OBu)_3_(OH) + BuOH
Condensation	Ti(OBu)_4_ + Ti(OBu)_3_(OH) → Ti_2_O(OBu)_6_ + BuOH
Dealcolation	2 Ti(OBu)_3_(OH) → Ti_2_O(OBu)_6_ + H_2_O
General reaction	Ti(OBu)_4_ + 2 H_2_O → TiO_2_ + 4 BuOH

## Data Availability

All data are included in the paper.

## Supplementary Materials

The following supporting information can be downloaded at: https://www.mdpi.com/article/10.3390/gels11060461/s1, Figure S1. Images of OP4 synthesis (a) after solvothermal treatment at 150 °C for 24 h and (b) after its vacuum drying; Figure S2. Raman spectra of anatase, with correspondence of the Raman peaks; Figure S3. UV/Vis spectra ofAg@TiO_2_ in ethanol, as synthetized (black line), after 1 week (blue line), after 2 weeks (red line) and after one month (green line); Figure S4. Dimensional distribution graphs for (a) Ag seeds and (b) Ag NPs for two-step synthesis; Figure S5. TEM images of two-step samples during the stages of the synthesis: (a,d) Ag NPs and Ag@TiO_2_ nanocomposites (b,e) before and (c,f) after solvothermal treatment. (c) Ag@TiO_2_ in ethanol treated at 150 °C for 24 h, (e) Ag@TiO_2_ treated in ethanol at 150 °C for 48 h; Table S1. Ag@TiO_2_ one-pot syntheses conditions and result of solvothermal treatment at 150 °C for 24 h; Table S2. Ag@TiO_2_ two-step syntheses condition for solvothermal treatment using a 23 mL Teflon liner vessel filled with 5 mL of suspension; Table S3. Comparison between the theoretical lattice spacings and the experimental values obtained by the analysis of the diffraction pattern. The diffraction patterns were analyzed using the DIFFRAC.EVA V6.0.0.9 software (Bruker AXS GmbH, Karlsruhe, Germany) in combination with the PDF-2 2002 database provided by the International Centre for Diffraction Data (ICDD).

## Abbreviations

The following abbreviations are used in this manuscript:

Ag NPs	Silver Nanoparticles
AS	Ag Seeds
BB	Bragg–Brentano
CCD	Charge-Coupled Device
CTAC	Cetiltrimethylammonium Chloride
CW	Continuous Wave
DMF	N,N-Dimethylformamide
EDS	Energy Dispersive X-ray Spectroscopy
EG	Ethylen Glicole
FWMH	Full Width at Half Maximum
GID	Grazing Incident Diffraction
HR-TEM	High-Resolution-Transmission Electron Microscopy
HAADF-STEM	High-Angle Annular Dark-Field Scanning Transmission Electron Microscopy
IR	Infrared
NIR	Near-Infrared
NPs	Nanoparticles
OP	One-Pot
OD	Optical Density
PTAs	Photothermal Agents
PTT	Photothermal Therapy
RCF	Relative Centrifugal Force
SAED	Selected Area Electron Diffraction
STEM	Scanning Transmission Electron Microscopy
TEM	Transmission Electron Microscopy
TTEAIP	Titanium-triethanolamine isopropoxide
TTIP	Titanium Tetraisopropoxide
TOB	Titanium Tetrabutoxide
UV/Vis	Ultraviolet/Visible
XRD	X-Ray Diffraction
