# Supplementary material for: Silver–Titania Nanocomposites for Photothermal Applications
Source: Gels. 2025 Jun 16;11(6):461. doi: 10.3390/gels11060461 (PMC12192153; doi:10.3390/gels11060461)
Supplement: Supplementary file 1 [file gels-11-00461-s001.zip › gels-3674727-supplementary.pdf]

gels

MDPI

Type of the Paper (Article, Review, Communication, etc.)

Silver-Titania nanocomposites for photothermal applications

Leonardo Bottacin[1], Roberto Zambon[1], Francesca Tajoli[1], Veronica Zani[1,2], Roberto Pilot[1,2], Naida El Habra[3], Silvia Gross[1,2], and Raffaella Signorini [1,2,*]

[1] Department of Chemical Science, University of Padua, Via Marzolo 1, I-35131 Padova, Italy; leonardo.bottacin@unipd.it (L.B.); roberto.zambon.2@phd.unipd.it (R.Z.); francesca.tajoli@unipd.it (F.T.); veronica.zani@unipd.it (V.Z.); roberto.pilot@unipd.it (R.P.); silvia.gross@unipd.it (S.G.)
[2] Consorzio Interuniversitario Nazionale per la Scienza e Tecnologia dei Materiali (INSTM), Via G. Giusti 9, I-50121 Firenze, Italy
[3] Istituto di Chimica della Materia Condensata e di Tecnologie per l'Energia (ICMATE), National Research Council (CNR), Corso Stati Uniti 4, I-35127 Padova, Italy; naida.elhabra@cnr.it
* Correspondence: raffaella.signorini@unipd.it; Tel.: +39-0498275118

Supporting Info

S.1 Samples and synthesis conditions

S.1.1 One-pot synthesis

Table S1. Ag@TiO_2 one-pot syntheses conditions and result of solvothermal treatment at 150°C for 24 h.

Samples	$AgNO_3$ [mg]	*Solution 1* [ml]	*Solution 2* [ml]	Molar Ratio TiO_2 : Ag	Reflux Time [min]	Color After Solvothermal
OP1	22	20	5	5.4:1	90	Black
OP2	21	60	15	5.7:1	90	Black
OP3	21	60	15	5.7:1	120	Colorless
OP4	22	100	25	5.3:1	100	Sand brown
OP5	22	60	15	5.3:1	90	Black
OP6	21	60	15	5.6:1	90	Black
OP7	23	100	25	5.1:1	90	Black/brown
OP8	23	100	25	5.1:1	90	Sand-brown
OP9	23	100	25	5.2:1	80	Colorless
OP10	23	100	25	5.0:1	90	Black
OP11	19	100	25	6.2:1	90	Brown

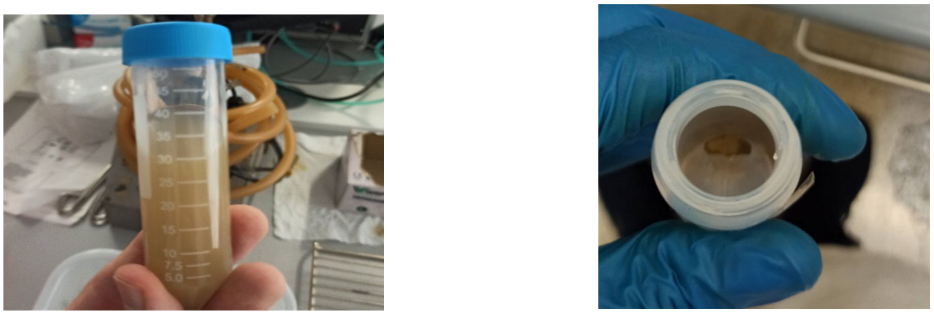

(**a**) (**b**)

Figure S1. Images of OP4 synthesis (a) after solvothermal treatment at 150°C for 24 h and (b) after its vacuum drying.

S.1.2 Two-step synthesis

Table S2. Ag@TiO_2 two-step syntheses condition for solvothermal treatment using a 23 mL Teflon liner vessel filled with 5 mL of suspension.

Sample	Time	Solvent	
	[hour]	% Ethanol	%water
Ag@TiO_2	24	100	0
	24	75	25
	24	25	75
	24	0	100
	48	100	0

S.2 Raman spectra mode

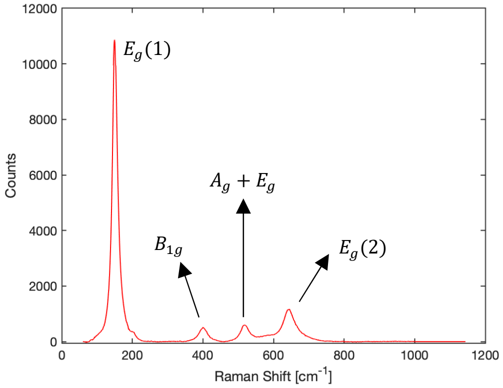

Figure S2. Raman spectra of anatase, with correspondence of the Raman peaks.

S.3 Phase match comparison for silver and anatase in XRD and HR-TEM analysis

Table S3. Comparison between the theoretical lattice spacings and the experimental values obtained by the analysis of the diffraction pattern. The diffraction patterns were analyzed using the DIFFRAC.EVA V6.0.0.9 software (Bruker AXS GmbH, Karlsruhe, Germany) in combination with the PDF-2 2002 database provided by the International Centre for Diffraction Data (ICDD).

	Experimental data	Literature data		
	(nm)	x (nm)	2ϑ (°)	[h k l]
Silver	0.234	0.236	38.121	[1 1 1]
	0.201	0.204	44.308	[2 0 0]
	0.144	0.144	64.458	[2 0 2]
Titania (anatase)	0.343	0.352	25.271	[0 1 1]
	0.231	0.234	38.509	[1 1 2]
	0.187	0.189	47.980	[0 2 0]
	0.164	0.167	54.994	[1 2 1]

S.4 Stability of nanosystems

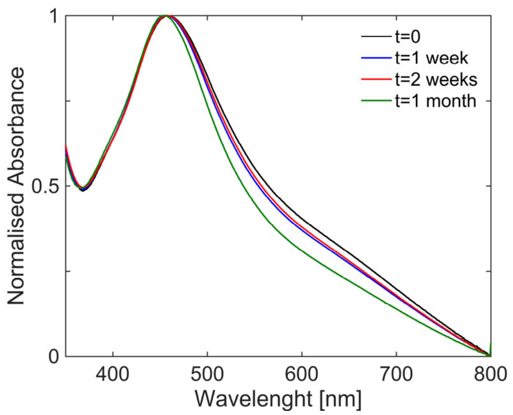

Figure S3. UV/Vis spectra ofAg@TiO_2 in ethanol, as synthetized (black line), after 1 week (blue line), after 2 weeks (red line) and after one month (green line).

S.5 TEM data

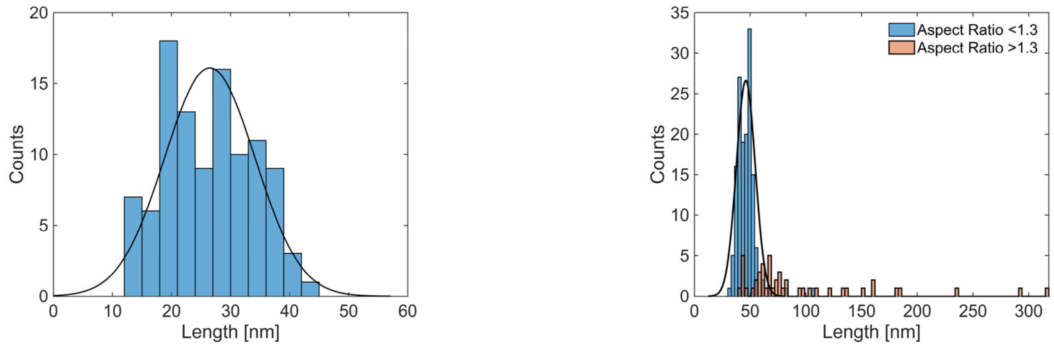

(**a**) (**b**)

Figure S4. Dimensional distribution graphs for (a) Ag seeds and (b) Ag NPs for two-step synthesis.

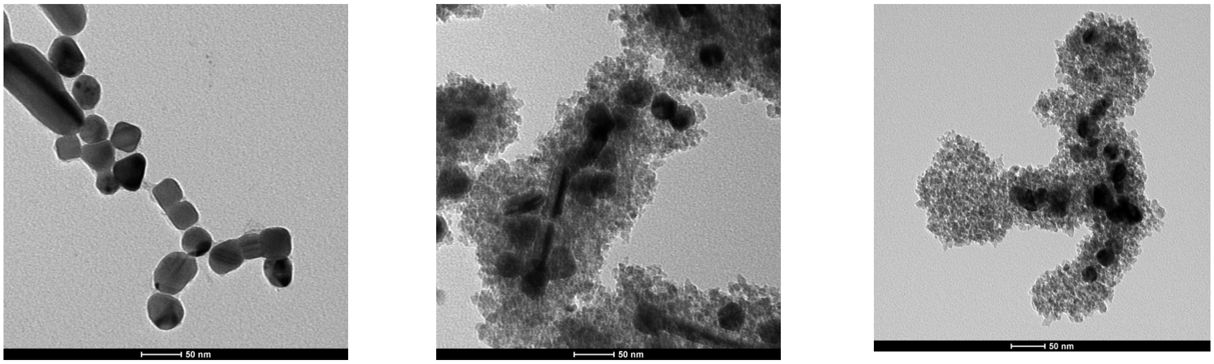

(**a**) (**b**) (**c**)

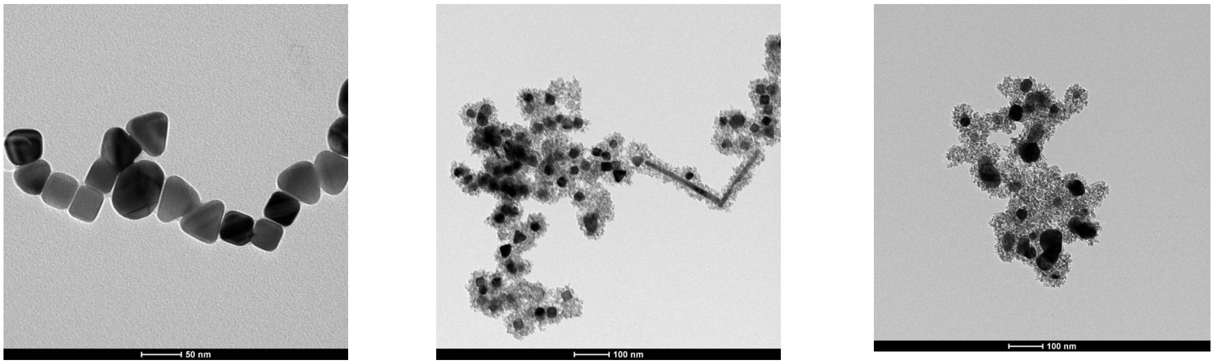

(d) **(e)** **(f)**

Figure S5. TEM images of two-step samples during the stages of the synthesis: (a, d) Ag NPs and Ag@TiO_2 nanocomposites (b, e) before and (c, f) after solvothermal treatment. (c) Ag@TiO_2 in ethanol treated at 150°C for 24h, (e) Ag@TiO_2 treated in ethanol at 150°C for 48h.
